# Crystal structure and Hirshfeld surface analysis of aqua­bis­(nicotinamide-κ*N*)bis­(4-sulfamoylbenzoato-κ*O* ^1^)copper(II)

**DOI:** 10.1107/S2056989017017765

**Published:** 2018-01-01

**Authors:** Tuncer Hökelek, Vijdan Yavuz, Hakan Dal, Hacali Necefoğlu

**Affiliations:** a Department of Physics, Hacettepe University, 06800 Beytepe, Ankara, Turkey; b Department of Chemistry, Kafkas University, 36100 Kars, Turkey; c Department of Chemistry, Anadolu University, 26470 Yenibağlar, Eskişehir, Turkey; d International Scientific Research Centre, Baku State University, 1148 Baku, Azerbaijan

**Keywords:** crystal structure, copper(II), transition metal complexes of benzoic acid and nicotinamide derivatives

## Abstract

In the crystal of the title complex, [Cu(C_7_H_6_NO_4_S)_2_(C_6_H_6_N_2_O)_2_(H_2_O)], the Cu^II^ cation and the O atom of the coordinated water mol­ecule reside on a twofold rotation axis. The Cu^II^ ion is coordinated by two carboxyl­ate O atoms of the two symmetry-related 4-sulfamoylbenzoate (SB) anions and by two N atoms of the two symmetry-related nicotinamide (NA) mol­ecules at distances of 1.978 (2) and 2.025 (3) Å, respectively, forming a slightly distorted square-planar arrangement. The distorted square-pyramidal coordination environment is completed by the water O atom in the axial position at a distance of 2.147 (4) Å. In the crystal, the mol­ecules are linked *via* O—H⋯O and N—H⋯O hydrogen bonds with *R*_2_^2^(8) and *R*_2_^2^(18) ring motifs, forming a three-dimensional architecture. The Hirshfeld surface analysis of the crystal structure indicates that the most important contributions for the crystal packing are from H⋯O/O⋯H (42.2%), H⋯H (25.7%) and H⋯C/C⋯H (20.0%) inter­actions.

## Abstract 2

The Cu^II^ cation, located on a twofold rotation axis, is coordinated by two 4-sulfamoylbenzoate anions, two nicotinamide (NA) mol­ecules and one water mol­ecule in a slightly distorted square-pyramidal geometry.

## Chemical context   

Nicotinamide (NA) is one form of niacin. A deficiency of this vitamin leads to loss of copper from the body, known as pellagra disease. Victims of pellagra show unusually high serum and urinary copper levels (Krishnamachari, 1974 ▸). The NA ring is the reactive part of nicotinamide adenine dinucleo­tide (NAD) and its phosphate (NADP), which are the major electron carriers in many biological oxidation-reduction reactions (You *et al.*, 1978 ▸). The nicotinic acid derivative *N*,*N*-di­ethyl­nicotinamide (DENA) is an important respiratory stimulant (Bigoli *et al.*, 1972 ▸).

Transition metal complexes with ligands of biochemical inter­est such as imidazole and some N-protected amino acids show inter­esting physical and/or chemical properties, through which they may find applications in biological systems (Antolini *et al.*, 1982 ▸). The crystal structures of metal complexes with benzoic acid derivatives have been reported extensively because of the varieties of the coordination modes, for example, Co and Cd complexes with 4-amino­benzoic acid (Chen & Chen, 2002 ▸). The structures of some mononuclear complexes obtained from the reactions of transition metal(II) ions with nicotinamide (NA) and some benzoic acid derivatives as ligands have been determined previously, *e.g.* [Zn(C_7_H_5_O_3_)_2_(C_6_H_6_N_2_O)_2_] [(II); Necefoğlu *et al.*, 2002 ▸], [Mn(C_7_H_4_ClO_2_)_2_(C_10_H_14_N_2_O)_2_(H_2_O)_2_] [(III); Hökelek *et al.*, 2008 ▸] and [Zn(C_7_H_4_BrO_2_)_2_(C_6_H_6_N_2_O)_2_(H_2_O)_2_] [(IV); Hökelek *et al.*, 2009 ▸].
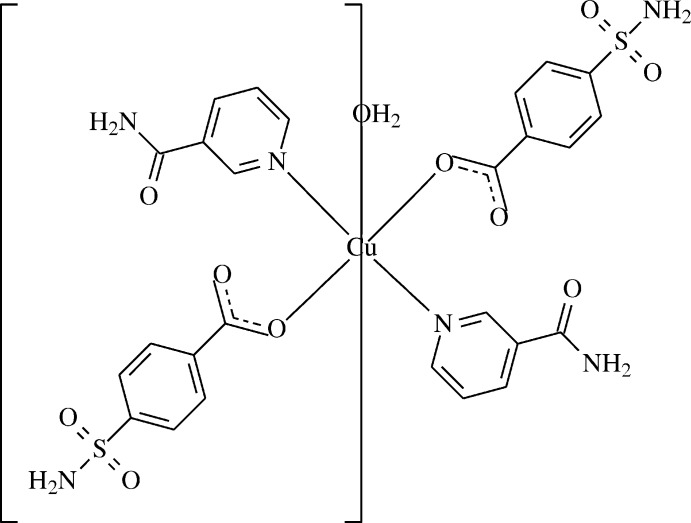


The structure determination of the title compound, (I), a copper complex with two 4-sulfamoylbenzoate (SB) anions and two nicotinamide (NA) ligands and one coordinated water mol­ecule, was undertaken in order to compare the results obtained with those reported previously. In this context, we synthesized the Cu^II^-containing title compound, aqua­bis(nicotinamide-κ*N*)bis­(4-sulfamoylbenzoato-κ*O^1^*)copper(II), [Cu(C_7_H_6_NO_4_S)_2_(C_6_H_6_N_2_O)_2_(H_2_O)], and report herein its crystal and mol­ecular structures along with the Hirshfeld surface analysis.

## Structural commentary   

**Figure 1:**
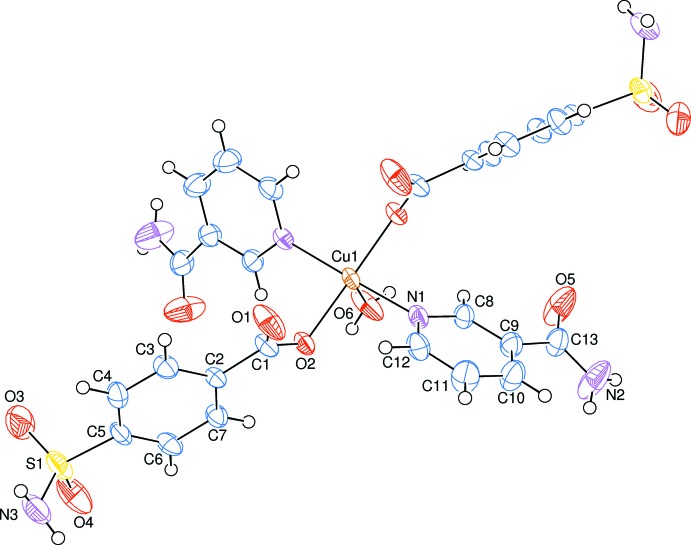
The mol­ecular structure of the title complex with the atom-numbering scheme. Unlabelled atoms are related to labelled ones by the symmetry operation 1 − *x*, *y*, 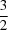 − *z*. Displacement ellipsoids are drawn at the 50% probability level.

The asymmetric unit of the crystal structure of the mononuclear title complex, (I), contains one half of the Cu^II^ ion, one 4-sulfamoylbenzoate (SB) anion and one nicotinamide (NA) mol­ecule together with one half water mol­ecule, all ligands coordinating in a monodentate manner (Fig. 1 ▸).

The Cu^II^ ion, located on a twofold rotation axis, is penta-coordinated *via* two nitro­gen atoms of NA and two oxygen atoms of SB anions and one oxygen atom of the water mol­ecule. The two carboxyl­ate O atoms [O2 and O2^i^; symmetry code: (i) 1 − *x*, *y*, 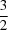 − *z*] of two symmetry-related monodentate SB anions and the two pyridine N atoms (N1 and N1^i^) of two symmetry-related monodentate NA ligands are at distances of 1.978 (2) and 2.025 (3) Å, respectively, from the Cu1 atom and form a slightly distorted square-planar arrangement. The sum of the bond angles N1—Cu1—O2^i^ [87.79 (10)°], N1^i^—Cu1—O2^i^ [92.08 (10)°], O2—Cu1—N1 [92.08 (10)°] and O2—Cu1—N1^i^ [87.79 (10)°] in the basal plane around Cu^II^ ion is 359.74°. This confirms the presence of Cu^II^ ion with very slight deviation from the basal plane. The slightly distorted square-pyramidal coordination environment is completed by the water O atom (O6) at a distance of 2.147 (4) Å in the axial position.

The near equalities of the C1—O1 [1.237 (4) Å] and C1—O2 [1.273 (4) Å] bonds in the carboxyl­ate groups indicate delocalized bonding arrangements, rather than localized single and double bonds. The O2—C1—O1 bond angle [125.2 (3)°] seems to be increased compared to that present in a free acid [122.2°]. The corresponding values for this angle in the closely related structures mentioned above are 123.5 (2) and 120.4 (2)° in (II), 125.2 (5)° in (III), and 124.3 (2)° in (IV); the benzoate ions are coordinated to the metal atoms only monodentately in (III) and (IV), and both monodentately and bidentately in (II). In the SB anion, the carboxyl­ate group is twisted away from the attached C2–C7 benzene ring by 20.92 (17)°, while the benzene and N1/C8–C12 pyridine rings are oriented at a dihedral angle of 81.86 (12)°.

## Supra­molecular features   

**Figure 2:**
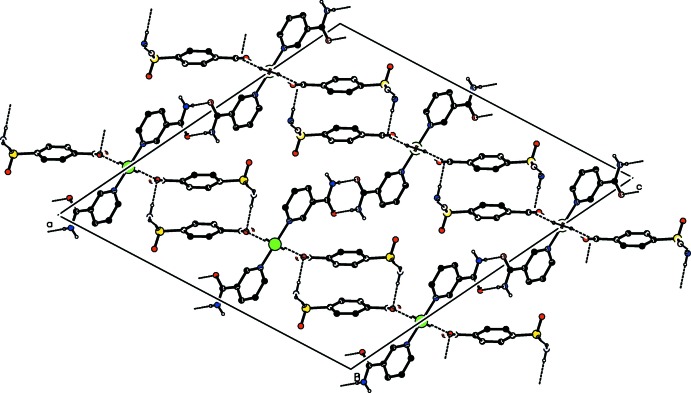
Part of the crystal structure, viewed down the *b* axis. O—H_W_⋯O_C_, N—H_NA_⋯O_NA_, N—H_SB_⋯O_C_ and N—H_SB_⋯O_SB_ (W = water, C = carboxyl­ate, NA = nicotinamide and SB = 4-sulfamoylbenzoate) hydrogen bonds, enclosing 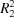
(8) and 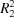
(18) ring motifs, are shown as dashed lines. H atoms not involved in these inter­actions have been omitted for clarity.

In the crystal, O—H_W_⋯O_C_, N—H_NA_⋯O_NA_, N—H_SB_⋯O_C_ and N—H_SB_⋯O_SB_ (W = water, C = carboxyl­ate, NA = nicotinamide and SB = 4-sulfamoylbenzoate) hydrogen bonds (Table 1 ▸) link the mol­ecules, enclosing 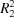
(8) and 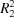
(18) ring motifs (Fig. 2 ▸) into a three-dimensional architecture. Hydrogen bonding and van der Waals contacts are the dominant inter­actions in the crystal packing. No significant π–π, C—H⋯π or C—H⋯O inter­actions are observed.

## Hirshfeld surface analysis   

**Figure 3:**
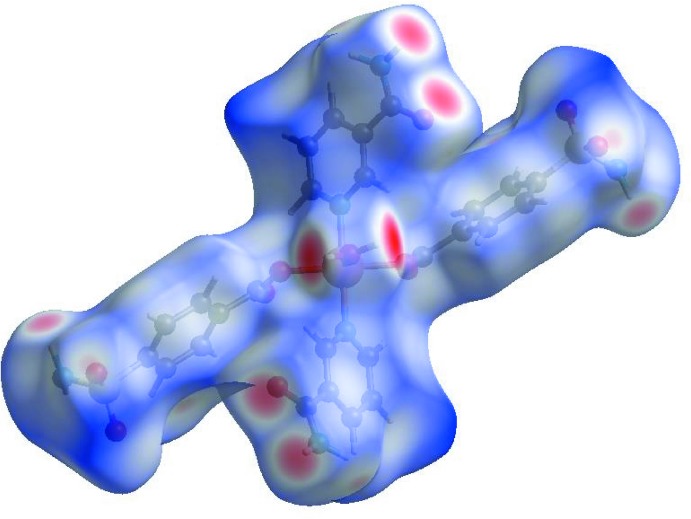
View of the three-dimensional Hirshfeld surface of the title complex plotted over *d*_norm_ in the range −0.7548 to 1.5398 a.u.

**Figure 4:**
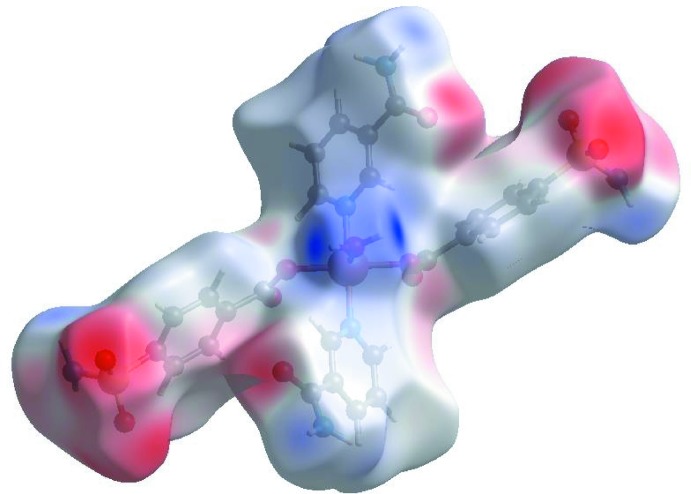
View of the three-dimensional Hirshfeld surface of the title complex plotted over electrostatic potential energy in the range −0.1045 to 0.2914 a.u. using the STO-3G basis set at the Hartree–Fock level of theory. N—H⋯O and O—H⋯O hydrogen-bond donors and acceptors are shown as blue and red regions around the atoms corresponding to positive and negative potentials, respectively.

**Figure 5:**
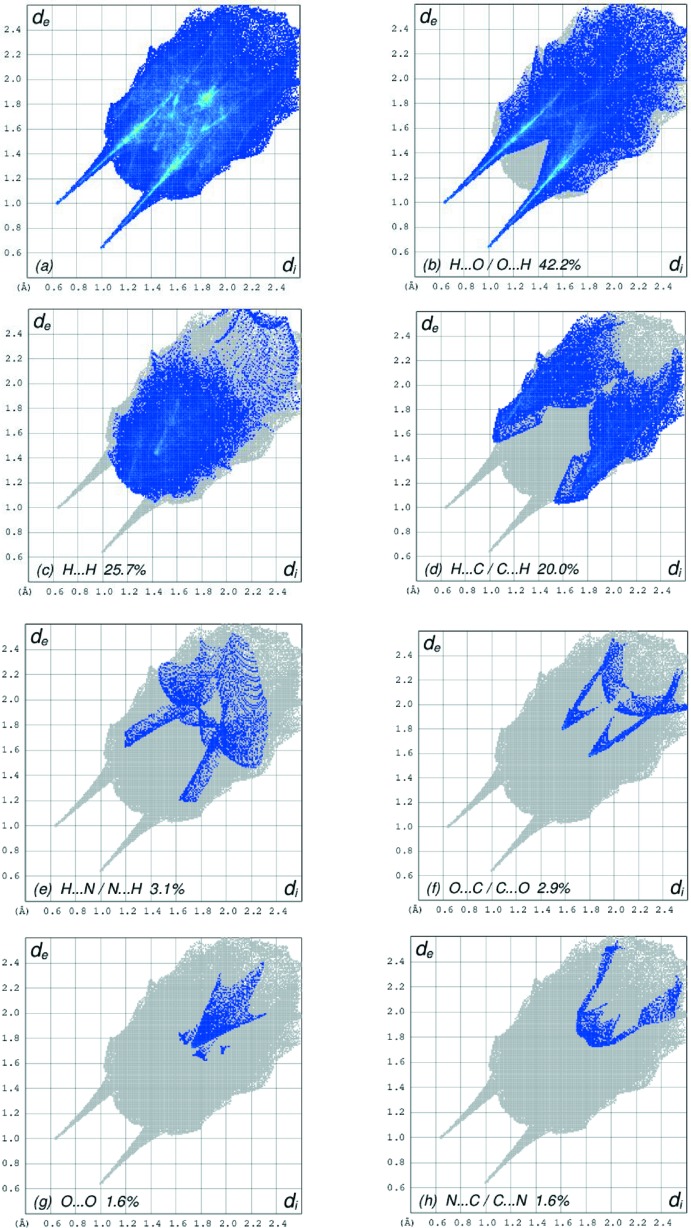
The full two-dimensional fingerprint plots for the title complex, showing (*a*) all inter­actions, and delineated into (*b*) H⋯O/O⋯H, (*c*) H⋯H, (*d*) H⋯C/C⋯H, (*e*) H⋯N/N⋯H, (*f*) O⋯C/C⋯O, (*g*) O⋯O and (*h*) N⋯C/C⋯N inter­actions. The *d*_i_ and *d*_e_ values are the closest inter­nal and external distances (in Å) from given points on the Hirshfeld surface contacts.

**Figure 6:**
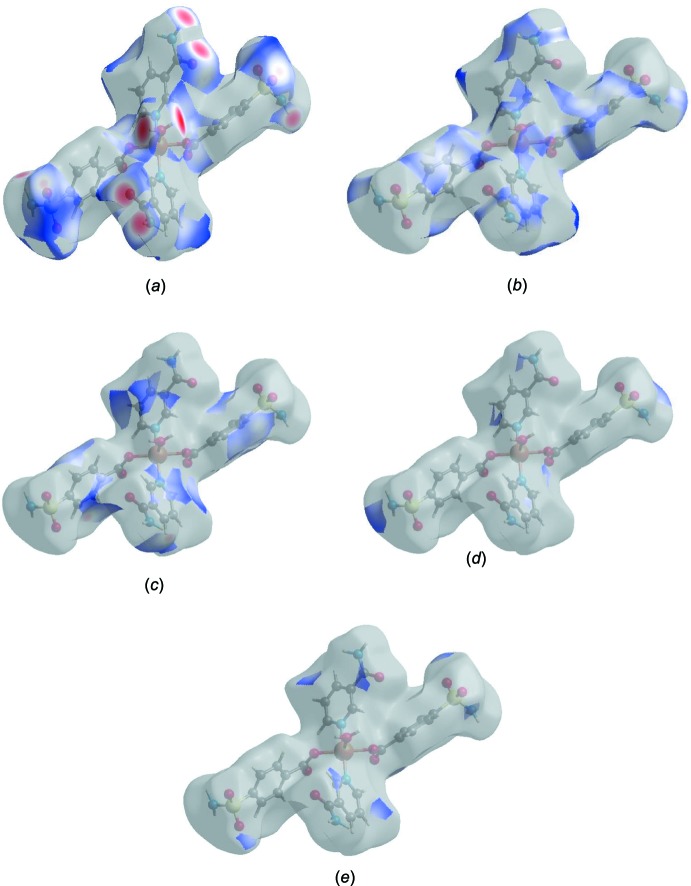
Representations of the Hirshfeld surface with the function *d*_norm_ plotted onto the surface for (*a*) H⋯O/O⋯H, (*b*) H⋯H, (*c*) H⋯C/C⋯H, (*d*) H⋯N/N⋯H and (*e*) O⋯C/C⋯O inter­actions.

In order to visualize the inter­molecular inter­actions in the crystal of the title complex, a Hirshfeld surface (HS) analysis (Hirshfeld, 1977 ▸; Spackman & Jayatilaka, 2009 ▸) was carried out by using *Crystal Explorer 17.5* (Turner *et al.*, 2017 ▸). In the HS plotted over *d*_norm_ (Fig. 3 ▸), the white surfaces indicate contacts with distances equal to the sum of van der Waals radii, and the red and blue colours indicate distances shorter (in close contact) or longer (distinct contact) than the van der Waals radii, respectively (Venkatesan *et al.*, 2016 ▸). The bright-red spots appearing near SB-O1, SB-O4, NA-O5 and hydrogen atoms H21, H31, H32 and H61 indicate their role as the respective donors and acceptors in the dominant O—H⋯O and N—H⋯O hydrogen bonds; they also appear as blue and red regions, respectively, corresponding to positive and negative potentials on the HS mapped over electrostatic potential (Spackman *et al.*, 2008 ▸; Jayatilaka *et al.*, 2005 ▸) as shown in Fig. 4 ▸. The blue regions indicate the positive electrostatic potential (hydrogen-bond donors), while the red regions indicate the negative electrostatic potential (hydrogen-bond acceptors). The overall two-dimensional fingerprint plot and those delineated into H⋯O/O⋯H, H⋯H, H⋯C/C⋯H, H⋯N/N⋯H, O⋯C/C⋯O, O⋯O and N⋯C/C⋯N contacts (McKinnon *et al.*, 2007 ▸) are illustrated in Fig. 5 ▸ *a*–*h*, respectively, together with their relative contributions to the Hirshfeld surface. The most important inter­action is H⋯O/O⋯H contributing 42.2% to the overall crystal packing, which is reflected in Fig. 5 ▸ *b* as a pair of spikes with the tip at *d*_e_ + *d*_i_ ∼1.63 Å. The short H⋯O/O⋯H contacts are masked by strong O—H⋯O hydrogen bonding in this plot. In the fingerprint plot delineated into H⋯H contacts (Fig. 5 ▸ *c*), the 25.7% contribution to the overall crystal packing is reflected as widely scattered points of high density due to the large hydrogen content of the mol­ecule. In the absence of C—H⋯π inter­actions in the crystal, the pair of characteristic wings resulting in the fingerprint plot delineated into H⋯C/C⋯H contacts with a 20.0% contribution to the HS, Fig. 5 ▸ *d*, and the pair of edges at *d*_e_ + *d*_i_ ∼2.58 Å result from short inter­atomic H⋯C/C⋯H contacts. The H⋯N/N⋯H (Fig. 5 ▸ *e*) and O⋯C/C⋯O (Fig. 5 ▸ *f*) contacts in the structure with 3.1% and 2.9% contributions to the HS have symmetrical distributions of points with the tips at *d*_e_ + *d*_i_ ∼2.8 Å and *d*_e_ + *d*_i_ ∼2.4 Å, arising from short inter­atomic H⋯N/N⋯H and O⋯C/C⋯O contacts, respectively. The O⋯O contacts assigned to short inter­atomic O⋯O contacts with a 1.6% contribution to the HS appear as an arrow-shaped distribution of points in Fig. 5 ▸ *g*, with the vertex at *d*_e_ = *d*_i_ ∼3.5 Å. Finally, the N⋯C/C⋯N contacts in the structure with a 1.6% contribution to the HS has a nearly symmetrical distribution of points, Fig. 5 ▸ *h*, with the scattered points of low density. The Hirshfeld surface representations with the function *d*_norm_ plotted onto the surface are shown for the H⋯O/O⋯H, H⋯H, H⋯C/C⋯H, H⋯N/N⋯H and O⋯C/C⋯O inter­actions in Fig. 6 ▸ *a*–*e*, respectively.

The Hirshfeld surface analysis confirms the importance of H-atom contacts in establishing the packing. The large number of H⋯O/O⋯H, H⋯H and H⋯C/C⋯H inter­actions suggest that van der Waals inter­actions and hydrogen bonding play the major roles in the crystal packing (Hathwar *et al.*, 2015 ▸).

## Synthesis and crystallization   

The title compound was prepared by the reaction of CuSO_4_·5H_2_O (1.25 g, 5 mmol) in H_2_O (100 ml) and nicotinamide (1.22 g, 10 mmol) in water (25 ml) with sodium 4-sulfamoylbenzoate (2.23 g, 10 mmol) in water (150 ml) at room temperature. The mixture was filtered and set aside for several days at ambient temperature to crystallize, giving blue single crystals (yield: 2.11 g, 29%). Combustion analysis: found; C, 42.85; H, 3.70; N, 11.68; S, 8.70%. Calculated: C_26_H_26_CuN_6_O_11_S_2_ C, 42.96; H, 3.58; N, 11.57; S, 8.81%. FT–IR: 3363, 3163, 1692, 1677, 1602, 1519, 1432, 1380, 1340, 1301, 1162, 1138, 1093, 1058, 778, 730, 688, 615, 532, 479, 427 cm^−1^.

## Refinement   

Crystal data, data collection and structure refinement details are summarized in Table 2 ▸. H atoms of the water mol­ecule and the NH_2_ group of the nicotinamide (NA) mol­ecule were located in a difference-Fourier map and refined freely. H atoms of the NH_2_ group of the 4-sulfomylbenzoate (SB) group were also located in a difference-Fourier map and the positions were refined with *U*_iso_(H) = 1.5*U*_eq_(N). The aromatic C-bound H atoms were positioned geometrically with C—H = 0.93 Å, and refined as riding with *U*_iso_(H) = 1.2*U*_eq_(C).

## Supplementary Material

Crystal structure: contains datablock(s) I, global. DOI: 10.1107/S2056989017017765/xu5914sup1.cif

Structure factors: contains datablock(s) I. DOI: 10.1107/S2056989017017765/xu5914Isup2.hkl

CCDC reference: 1810805

Additional supporting information:  crystallographic information; 3D view; checkCIF report

## Figures and Tables

**Table 1:** Hydrogen-bond geometry (Å, °)

*D*—H⋯*A*	*D*—H	H⋯*A*	*D*⋯*A*	*D*—H⋯*A*
N2—H21⋯O5^i^	0.78 (5)	2.23 (5)	3.009 (7)	176 (5)
N3—H31⋯O1^ii^	0.88 (7)	2.33 (7)	3.165 (6)	159 (6)
N3—H32⋯O4^iii^	0.93 (6)	2.54 (6)	3.452 (6)	165 (6)
O6—H61⋯O1^iv^	0.83 (5)	1.79 (5)	2.603 (4)	167 (4)

Symmetry codes: (i) 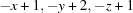; (ii) 
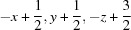
; (iii) 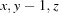; (iv) 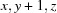.

**Table 2:** Experimental details

Crystal data	
Chemical formula	[Cu(C_7_H_6_NO_4_S)_2_(C_6_H_6_N_2_O)_2_(H_2_O)]
*M*_r_	726.20
Crystal system, space group	Monoclinic, *C*2/*c*
Temperature (K)	296
*a*, *b*, *c* (Å)	24.2353 (4), 5.6080 (2), 24.9702 (4)
β (°)	118.027 (11)
*V* (Å^3^)	2995.7 (3)
*Z*	4
Radiation type	Mo *K*α
μ (mm^−1^)	0.94
Crystal size (mm)	0.22 × 0.17 × 0.15

Data collection	
Diffractometer	Bruker APEXII CCD
Absorption correction	Multi-scan (*SADABS*; Bruker, 2012 ▸)
*T*_min_, *T*_max_	0.812, 0.853
No. of measured, independent and observed [*I* > 2σ(*I*)] reflections	26534, 3069, 2535
*R*_int_	0.049
(sin θ/λ)_max_ (Å^−1^)	0.627

Refinement	
*R*[*F* ^2^ > 2σ(*F* ^2^)], *wR*(*F* ^2^), *S*	0.048, 0.109, 1.10
No. of reflections	3069
No. of parameters	227
H-atom treatment	H atoms treated by a mixture of independent and constrained refinement
Δρ_max_, Δρ_min_ (e Å^−3^)	0.50, −0.58

Computer programs: *APEX2* and *SAINT* (Bruker, 2012 ▸), *SHELXS97* and *SHELXL97* (Sheldrick, 2008 ▸), *ORTEP-3 for Windows* and *WinGX* (Farrugia, 2012 ▸) and *PLATON* (Spek, 2015 ▸).

## supplementary crystallographic information

### Crystal data

**Table:**

[Cu(C_7_H_6_NO_4_S)_2_(C_6_H_6_N_2_O)_2_(H_2_O)]	*F*(000) = 1492
*M**_r_* = 726.20	*D*_x_ = 1.610 Mg m^−^^3^
Monoclinic, *C*2/*c*	Mo *K*α radiation, λ = 0.71073 Å
Hall symbol: -C 2yc	Cell parameters from 9671 reflections
*a* = 24.2353 (4) Å	θ = 3.2–26.5°
*b* = 5.6080 (2) Å	µ = 0.94 mm^−^^1^
*c* = 24.9702 (4) Å	*T* = 296 K
β = 118.027 (11)°	Block, blue
*V* = 2995.7 (3) Å^3^	0.22 × 0.17 × 0.15 mm
*Z* = 4	

### Data collection

**Table:**

Bruker APEXII CCD diffractometer	3069 independent reflections
Radiation source: fine-focus sealed tube	2535 reflections with *I* > 2σ(*I*)
Graphite monochromator	*R*_int_ = 0.049
φ and ω scans	θ_max_ = 26.5°, θ_min_ = 3.2°
Absorption correction: multi-scan (SADABS; Bruker, 2012)	*h* = −30→30
*T*_min_ = 0.812, *T*_max_ = 0.853	*k* = −6→7
26534 measured reflections	*l* = −31→31

### Refinement

**Table:**

Refinement on *F*^2^	Primary atom site location: structure-invariant direct methods
Least-squares matrix: full	Secondary atom site location: difference Fourier map
*R*[*F*^2^ > 2σ(*F*^2^)] = 0.048	Hydrogen site location: inferred from neighbouring sites
*wR*(*F*^2^) = 0.109	H atoms treated by a mixture of independent and constrained refinement
*S* = 1.10	*w* = 1/[σ^2^(*F*_o_^2^) + (0.0186*P*)^2^ + 15.4435*P*] where *P* = (*F*_o_^2^ + 2*F*_c_^2^)/3
3069 reflections	(Δ/σ)_max_ < 0.001
227 parameters	Δρ_max_ = 0.50 e Å^−^^3^
0 restraints	Δρ_min_ = −0.58 e Å^−^^3^

### Special details

**Table:**

Geometry. All esds (except the esd in the dihedral angle between two l.s. planes) are estimated using the full covariance matrix. The cell esds are taken into account individually in the estimation of esds in distances, angles and torsion angles; correlations between esds in cell parameters are only used when they are defined by crystal symmetry. An approximate (isotropic) treatment of cell esds is used for estimating esds involving l.s. planes.
Refinement. Refinement of F^2^ against ALL reflections. The weighted R-factor wR and goodness of fit S are based on F^2^, conventional R-factors R are based on F, with F set to zero for negative F^2^. The threshold expression of F^2^ > 2sigma(F^2^) is used only for calculating R-factors(gt) etc. and is not relevant to the choice of reflections for refinement. R-factors based on F^2^ are statistically about twice as large as those based on F, and R- factors based on ALL data will be even larger.

### Fractional atomic coordinates and isotropic or equivalent isotropic displacement parameters (Å^2^)

**Table:**

	*x*	*y*	*z*	*U*_iso_*/*U*_eq_	
Cu1	0.5000	0.37776 (10)	0.7500	0.02603 (16)	
S1	0.24180 (5)	0.3629 (2)	0.89016 (5)	0.0522 (3)	
O1	0.40911 (14)	−0.0206 (5)	0.75744 (16)	0.0598 (9)	
O2	0.41703 (10)	0.3706 (4)	0.74704 (10)	0.0324 (5)	
O3	0.28139 (15)	0.2819 (9)	0.95048 (13)	0.0852 (13)	
O4	0.22142 (16)	0.6045 (7)	0.87921 (18)	0.0791 (11)	
O5	0.52585 (16)	0.8045 (7)	0.56765 (14)	0.0885 (13)	
O6	0.5000	0.7606 (7)	0.7500	0.0580 (13)	
H61	0.475 (2)	0.848 (8)	0.755 (2)	0.066 (15)*	
N1	0.46156 (12)	0.3576 (5)	0.65848 (12)	0.0310 (6)	
N2	0.4387 (3)	0.7680 (9)	0.48231 (18)	0.0793 (16)	
H21	0.449 (2)	0.875 (10)	0.469 (2)	0.073 (17)*	
H22	0.402 (3)	0.725 (14)	0.465 (3)	0.14 (3)*	
N3	0.18052 (17)	0.1999 (9)	0.86238 (19)	0.0613 (11)	
H31	0.153 (3)	0.239 (11)	0.825 (3)	0.092*	
H32	0.191 (3)	0.041 (11)	0.874 (3)	0.092*	
C1	0.39732 (15)	0.1892 (6)	0.76359 (16)	0.0330 (7)	
C2	0.35674 (14)	0.2360 (6)	0.79293 (14)	0.0268 (6)	
C3	0.35135 (16)	0.0600 (6)	0.82907 (16)	0.0349 (8)	
H3	0.3721	−0.0843	0.8340	0.042*	
C4	0.31532 (16)	0.0973 (7)	0.85791 (16)	0.0391 (8)	
H4	0.3121	−0.0201	0.8826	0.047*	
C5	0.28411 (15)	0.3119 (7)	0.84941 (15)	0.0348 (8)	
C6	0.28860 (16)	0.4881 (7)	0.81305 (16)	0.0379 (8)	
H6	0.2667	0.6303	0.8071	0.045*	
C7	0.32605 (15)	0.4514 (6)	0.78542 (16)	0.0344 (8)	
H7	0.3305	0.5712	0.7620	0.041*	
C8	0.47946 (16)	0.5133 (6)	0.62909 (15)	0.0328 (7)	
H8	0.5116	0.6192	0.6518	0.039*	
C9	0.45234 (16)	0.5243 (7)	0.56628 (15)	0.0368 (8)	
C10	0.40550 (18)	0.3622 (8)	0.53334 (17)	0.0499 (10)	
H10	0.3865	0.3632	0.4912	0.060*	
C11	0.38710 (19)	0.1995 (8)	0.56309 (18)	0.0523 (10)	
H11	0.3558	0.0892	0.5414	0.063*	
C12	0.41574 (16)	0.2027 (7)	0.62562 (16)	0.0392 (8)	
H12	0.4029	0.0940	0.6457	0.047*	
C13	0.47507 (18)	0.7119 (7)	0.53885 (16)	0.0433 (9)	

### Atomic displacement parameters (Å^2^)

**Table:**

	*U*^11^	*U*^22^	*U*^33^	*U*^12^	*U*^13^	*U*^23^
Cu1	0.0283 (3)	0.0251 (3)	0.0346 (3)	0.000	0.0230 (2)	0.000
S1	0.0401 (5)	0.0787 (8)	0.0529 (6)	−0.0098 (5)	0.0344 (5)	−0.0187 (6)
O1	0.0730 (19)	0.0313 (15)	0.112 (3)	0.0088 (14)	0.074 (2)	−0.0004 (16)
O2	0.0299 (11)	0.0354 (13)	0.0409 (12)	−0.0012 (10)	0.0242 (10)	0.0018 (11)
O3	0.061 (2)	0.159 (4)	0.0415 (16)	−0.010 (2)	0.0294 (15)	−0.012 (2)
O4	0.079 (2)	0.076 (2)	0.118 (3)	−0.0090 (19)	0.076 (2)	−0.032 (2)
O5	0.077 (2)	0.113 (3)	0.0510 (18)	−0.054 (2)	0.0096 (17)	0.0285 (19)
O6	0.062 (3)	0.0222 (19)	0.125 (4)	0.000	0.073 (3)	0.000
N1	0.0304 (13)	0.0338 (15)	0.0363 (14)	−0.0026 (12)	0.0218 (12)	0.0023 (12)
N2	0.088 (3)	0.082 (3)	0.041 (2)	−0.037 (3)	0.007 (2)	0.024 (2)
N3	0.042 (2)	0.086 (3)	0.070 (2)	−0.011 (2)	0.0388 (19)	−0.007 (2)
C1	0.0293 (16)	0.0304 (18)	0.0480 (19)	−0.0003 (14)	0.0253 (15)	0.0002 (15)
C2	0.0242 (14)	0.0255 (16)	0.0349 (16)	−0.0009 (13)	0.0173 (13)	−0.0004 (13)
C3	0.0351 (17)	0.0266 (17)	0.048 (2)	0.0040 (14)	0.0236 (16)	0.0042 (15)
C4	0.0406 (19)	0.042 (2)	0.0438 (19)	−0.0010 (16)	0.0271 (17)	0.0084 (17)
C5	0.0261 (16)	0.048 (2)	0.0380 (18)	−0.0042 (15)	0.0215 (15)	−0.0063 (16)
C6	0.0343 (17)	0.0341 (19)	0.053 (2)	0.0072 (15)	0.0268 (17)	−0.0018 (17)
C7	0.0366 (18)	0.0305 (17)	0.0442 (19)	0.0062 (14)	0.0257 (16)	0.0064 (15)
C8	0.0365 (17)	0.0331 (18)	0.0324 (17)	−0.0041 (15)	0.0191 (14)	0.0028 (14)
C9	0.0379 (18)	0.0387 (19)	0.0351 (18)	−0.0036 (16)	0.0183 (15)	0.0030 (16)
C10	0.049 (2)	0.062 (3)	0.0333 (18)	−0.017 (2)	0.0154 (17)	−0.0004 (19)
C11	0.052 (2)	0.058 (3)	0.044 (2)	−0.023 (2)	0.0201 (19)	−0.007 (2)
C12	0.0397 (19)	0.040 (2)	0.046 (2)	−0.0100 (16)	0.0270 (17)	0.0002 (17)
C13	0.050 (2)	0.046 (2)	0.0348 (19)	−0.0064 (18)	0.0214 (17)	0.0045 (17)

### Geometric parameters (Å, º)

**Table:**

Cu1—O2	1.978 (2)	C1—C2	1.500 (4)
Cu1—O2^i^	1.978 (2)	C2—C7	1.384 (4)
Cu1—N1	2.025 (3)	C2—C3	1.384 (5)
Cu1—N1^i^	2.025 (3)	C3—C4	1.384 (5)
Cu1—O6	2.147 (4)	C3—H3	0.9300
S1—O4	1.424 (4)	C4—C5	1.384 (5)
S1—O3	1.428 (3)	C4—H4	0.9300
S1—N3	1.598 (4)	C5—C6	1.380 (5)
S1—C5	1.775 (3)	C6—C7	1.389 (5)
O1—C1	1.237 (4)	C6—H6	0.9300
O2—C1	1.273 (4)	C7—H7	0.9300
O5—C13	1.212 (5)	C8—C9	1.388 (5)
O6—H61	0.83 (4)	C8—H8	0.9300
N1—C8	1.337 (4)	C9—C10	1.383 (5)
N1—C12	1.344 (4)	C9—C13	1.495 (5)
N2—C13	1.303 (5)	C10—C11	1.377 (5)
N2—H21	0.78 (6)	C10—H10	0.9300
N2—H22	0.82 (7)	C11—C12	1.379 (5)
N3—H31	0.88 (5)	C11—H11	0.9300
N3—H32	0.93 (6)	C12—H12	0.9300
			
O2—Cu1—O2^i^	177.67 (14)	C4—C3—C2	120.5 (3)
O2—Cu1—N1	92.08 (10)	C4—C3—H3	119.8
O2^i^—Cu1—N1	87.79 (10)	C2—C3—H3	119.8
O2—Cu1—N1^i^	87.79 (10)	C5—C4—C3	119.0 (3)
O2^i^—Cu1—N1^i^	92.08 (10)	C5—C4—H4	120.5
N1—Cu1—N1^i^	173.61 (16)	C3—C4—H4	120.5
O2—Cu1—O6	91.16 (7)	C6—C5—C4	121.1 (3)
O2^i^—Cu1—O6	91.16 (7)	C6—C5—S1	120.5 (3)
N1—Cu1—O6	93.20 (8)	C4—C5—S1	118.3 (3)
N1^i^—Cu1—O6	93.20 (8)	C5—C6—C7	119.5 (3)
O4—S1—O3	120.4 (3)	C5—C6—H6	120.3
O4—S1—N3	107.1 (2)	C7—C6—H6	120.3
O3—S1—N3	107.6 (2)	C2—C7—C6	119.8 (3)
O4—S1—C5	106.55 (19)	C2—C7—H7	120.1
O3—S1—C5	105.69 (18)	C6—C7—H7	120.1
N3—S1—C5	109.17 (19)	N1—C8—C9	123.1 (3)
C1—O2—Cu1	122.3 (2)	N1—C8—H8	118.5
Cu1—O6—H61	126 (3)	C9—C8—H8	118.5
C8—N1—C12	118.4 (3)	C10—C9—C8	117.6 (3)
C8—N1—Cu1	119.1 (2)	C10—C9—C13	124.5 (3)
C12—N1—Cu1	122.3 (2)	C8—C9—C13	117.9 (3)
C13—N2—H21	117 (4)	C11—C10—C9	119.9 (3)
C13—N2—H22	122 (5)	C11—C10—H10	120.1
H21—N2—H22	118 (6)	C9—C10—H10	120.1
S1—N3—H31	113 (4)	C10—C11—C12	119.0 (4)
S1—N3—H32	110 (4)	C10—C11—H11	120.5
H31—N3—H32	122 (6)	C12—C11—H11	120.5
O1—C1—O2	125.2 (3)	N1—C12—C11	122.1 (3)
O1—C1—C2	118.0 (3)	N1—C12—H12	118.9
O2—C1—C2	116.8 (3)	C11—C12—H12	118.9
C7—C2—C3	120.1 (3)	O5—C13—N2	121.3 (4)
C7—C2—C1	121.4 (3)	O5—C13—C9	121.3 (3)
C3—C2—C1	118.6 (3)	N2—C13—C9	117.4 (4)
			
N1—Cu1—O2—C1	107.8 (3)	O4—S1—C5—C4	172.7 (3)
N1^i^—Cu1—O2—C1	−65.8 (3)	O3—S1—C5—C4	43.5 (4)
O6—Cu1—O2—C1	−158.9 (2)	N3—S1—C5—C4	−72.0 (3)
O2—Cu1—N1—C8	130.0 (3)	C4—C5—C6—C7	−1.3 (5)
O2^i^—Cu1—N1—C8	−52.3 (3)	S1—C5—C6—C7	174.9 (3)
O6—Cu1—N1—C8	38.8 (2)	C3—C2—C7—C6	−1.6 (5)
O2—Cu1—N1—C12	−45.7 (3)	C1—C2—C7—C6	179.7 (3)
O2^i^—Cu1—N1—C12	132.0 (3)	C5—C6—C7—C2	2.2 (5)
O6—Cu1—N1—C12	−137.0 (3)	C12—N1—C8—C9	0.9 (5)
Cu1—O2—C1—O1	−33.8 (5)	Cu1—N1—C8—C9	−175.0 (3)
Cu1—O2—C1—C2	146.0 (2)	N1—C8—C9—C10	−1.3 (6)
O1—C1—C2—C7	−160.2 (4)	N1—C8—C9—C13	178.0 (3)
O2—C1—C2—C7	19.9 (5)	C8—C9—C10—C11	0.6 (6)
O1—C1—C2—C3	21.1 (5)	C13—C9—C10—C11	−178.6 (4)
O2—C1—C2—C3	−158.8 (3)	C9—C10—C11—C12	0.4 (7)
C7—C2—C3—C4	0.1 (5)	C8—N1—C12—C11	0.2 (5)
C1—C2—C3—C4	178.8 (3)	Cu1—N1—C12—C11	176.0 (3)
C2—C3—C4—C5	0.8 (5)	C10—C11—C12—N1	−0.8 (6)
C3—C4—C5—C6	−0.2 (5)	C10—C9—C13—O5	−159.7 (5)
C3—C4—C5—S1	−176.5 (3)	C8—C9—C13—O5	21.1 (6)
O4—S1—C5—C6	−3.5 (4)	C10—C9—C13—N2	18.7 (7)
O3—S1—C5—C6	−132.7 (3)	C8—C9—C13—N2	−160.4 (4)
N3—S1—C5—C6	111.8 (3)		

Symmetry code: (i) −*x*+1, *y*, −*z*+3/2.

### Hydrogen-bond geometry (Å, º)

**Table:**

*D*—H···*A*	*D*—H	H···*A*	*D*···*A*	*D*—H···*A*
N2—H21···O5^ii^	0.78 (5)	2.23 (5)	3.009 (7)	176 (5)
N3—H31···O1^iii^	0.88 (7)	2.33 (7)	3.165 (6)	159 (6)
N3—H32···O4^iv^	0.93 (6)	2.54 (6)	3.452 (6)	165 (6)
O6—H61···O1^v^	0.83 (5)	1.79 (5)	2.603 (4)	167 (4)

Symmetry codes: (ii) −*x*+1, −*y*+2, −*z*+1; (iii) −*x*+1/2, *y*+1/2, −*z*+3/2; (iv) *x*, *y*−1, *z*; (v) *x*, *y*+1, *z*.
